# Transition between strong and weak topological insulator in ZrTe_5_ and HfTe_5_

**DOI:** 10.1038/srep45667

**Published:** 2017-04-04

**Authors:** Zongjian Fan, Qi-Feng Liang, Y. B. Chen, Shu-Hua Yao, Jian Zhou

**Affiliations:** 1 National Laboratory of Solid State Microstructures and Department of Materials Science and Engineering, Nanjing University, Nanjing 210093, China; 2 Department of Physics, Shaoxing University, Shaoxing 312000, China; 3 National Laboratory of Solid State Microstructures and Department of Physics, Nanjing University, Nanjing 210093, China; 4 Collaborative Innovation Center of Advanced Microstructures, Nanjing University, Nanjing, 210093, China

## Abstract

ZrTe_5_ and HfTe_5_ have attracted increasingly attention recently since the theoretical prediction of being topological insulators (TIs). However, subsequent works show many contradictions about their topolog-ical nature. Three possible phases, i.e. strong TI, weak TI, and Dirac semi-metal, have been observed in different experiments until now. Essentially whether ZrTe_5_ or HfTe_5_ has a band gap or not is still a question. Here, we present detailed first-principles calculations on the electronic and topological prop-erties of ZrTe_5_ and HfTe_5_ on variant volumes and clearly demonstrate the topological phase transition from a strong TI, going through an intermediate Dirac semi-metal state, then to a weak TI when the crystal expands. Our work might give a unified explain about the divergent experimental results and propose the crucial clue to further experiments to elucidate the topological nature of these materials.

Topological insulator (TI) is a new class of material which is an insulator in its bulk, while having time reversal symmetry protected conducting states on the edge or surface^1^,^2^,^3^. A large number of realistic materials have been theoretically proposed and experimentally confirmed, such as Bi_2_Se_3_ and Bi_2_Te_3_^4^,^5^. However, the layered transition-metal pentatelluride ZrTe_5_ and HfTe_5_ is a particular example. ZrTe_5_ and HfTe_5_ were studied more than 30 years ago due to the large thermoelectric power^6^ and mysterious resistivity anomaly^7^,^8^. Recently, Weng *et al*. predicted that mono-layer ZrTe_5_ and HfTe_5_ are good quantum spin Hall insulators with relatively large bulk band gap (about 0.1 eV) by first principles calculations^9^. The three-dimensional (3D) bulk phase of ZrTe_5_ and HfTe_5_ are also predicted to be TIs, which are located at the vicinity of a transition between strong and weak TI, but without detailed description^9^.

Nevertheless, subsequent experiments show many contradictions about the topological nature of ZrTe_5_ or HfTe_5_. Several experimental works suggested that ZrTe_5_ is a Dirac semi-metal without a finite band gap by different characterization methods, such as Shubnikov-de Haas oscillations, angle-resolved photoemission spectroscopy (ARPES), and infrared reflectivity measurements^10^,^11^,^12^,^13^,^14^,^15^. Of course there are also other experimental works holding opposite point of view. For example, in two recent scanning tunneling microscopy (STM) experiments, they unambiguously observed a large bulk band gap about 80 or 100 meV in ZrTe_5_^16^,^17^, implying that there is no surface state on the top surface and therefore ZrTe_5_ should be a weak TI. Another APRES work also favored a weak TI for ZrTe_5_^18^. However, there are two other ARPES works which believed that ZrTe_5_ is a strong TI^19^,^20^. For instance, by using the comprehensive ARPES, STM, and first principles calculations, Manzoni *et al*. found a metallic density of state (DOS) at Fermi energy, which arises from the two-dimensional surface state and thus indicates ZrTe_5_ is a strong TI^19^.

The divergence of these experiments make ZrTe_5_ (HfTe_5_) being a very puzzling but interesting material, which needs more further experimental and theoretical studies. Therefore, in order to figure out the physical mechanism behind those contradictory experimental results, we revisited the band structures of ZrTe_5_ and HfTe_5_, and carefully studied their relationship with the volume expansion. We find a clear topological transition between a strong and weak TI in ZrTe_5_ and HfTe_5_, accompanied by an intermediate Dirac semi-metal state between them. This work could shed more light on a unified explain about the different experimental results, and propose the crucial clue to further experiments to elucidate the topological nature of ZrTe_5_ and HfTe_5_.

## Results

**Figure 1:**
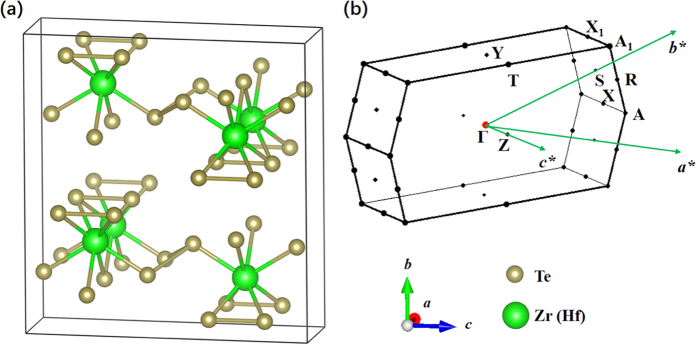
(**a**) Layered crystal stucture of ZrTe_5_ (HfTe_5_) in the orthorhombic conventional unit cell. Big green and small brown balls represent Zr (Hf) and Te atoms respectively. The layers stack along the *b* direction. (**b**) Brillouin zone and the high symmetry points of the primitive unit cell of ZrTe_5_ (HfTe_5_).

As shown in Fig. 1(a), ZrTe_5_ and HfTe_5_ share the same base-centered orthorhombic crystal structure with Cmcm (No. 63) space group symmetry. Trigonal prismatic ZrTe_3_ chains oriented along the *a* axis make up the ZrTe_5_ natural cleavage plane. Each chain consists of one Zr atom and two different kinds of Te atoms. ZrTe_3_ chains are connected by zigzag Te chains along the *c* axis, building a two dimensional structure of ZrTe_5_ in the *a*-*c* plane. One crystal unit cell contains two ZrTe_5_ planes piled along the *b* axis, the stacking orientation of ZrTe_5_. The Brillouin zone and high symmetry k-points of ZrTe_5_ (HfTe_5_) are shown in Fig. 1(b), in which a^*^, b^*^, and c^*^ are the reciprocal lattice vectors.

Due to the weak the van der Waals (vdw) interaction in the layer ZrTe_5_ (HfTe_5_)^9^, the vdw corrected correlation functional is necessary in order to obtain good theoretical lattice constants. In Table 1, we present the optimized lattice constants of ZrTe_5_ and HfTe_5_ based on the optB86b-vdw functional, as well as the experimental ones^21^. We find that the theoretical and experimental lattice constants are well consistent with each other and the maximum difference between them is less than 1%. For comparison, we also optimized the structures of ZrTe_5_ and HfTe_5_ by using the standard Perdew-Burke-Ernzerhof (PBE) exchange correlation^22^. It is obvious that there is a large error (about 9%) in the lattice *b* (in the stacking direction), which indicates that standard PBE failed to describe the structures of ZrTe_5_ andHfTe_5_, and the vdw correction is necessary.

**Figure 2:**
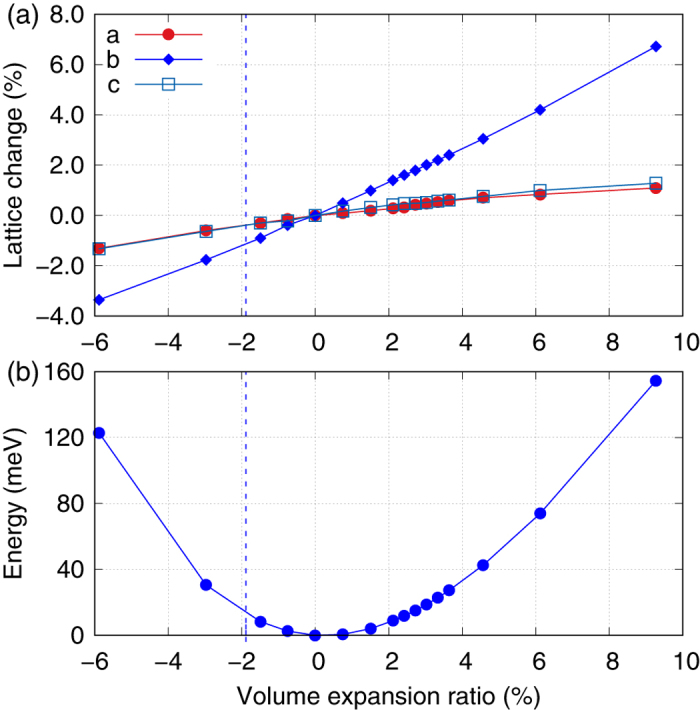
(**a**) Lattice constants *a, b*, and *c* under different unit cell volumes. (**b**) Relative total energy of a primitive unit cell of ZrTe_5_ under different volumes. Blue vertical dotted lines represent the experimental volume at 10 K^21^.

In order to explore the possible topological phase transition in ZrTe_5_ (HfTe_5_), we then study their electronic properties under different volumes. Based on the above optimized structure, we change volume of the unit cell by hand and then optimize the atom positions and lattice constants under each variant volumes. This process can simulate the hydrostatic pressure experiments or the thermal expansion effect due to finite temperatures. It is noted that we did not change the volume drastically and the system is far away from the region of superconductivity phase under high pressure found in ZrTe_5_ and HfTe_5_^23^,^24^,^25^. In Fig. 2(a), we present the change of lattice constants *a, b*, and *c* under different volume expansion ratios defined as (*V* − *V*_0_)/*V*_0_ × 100%, where *V*_0_ is the unit cell volume at theoretical ground state listed in the Table 1. It is found that all the lattice constants have similar linear dependence on the volume of the unit cell. But the in-plane lattice constants *a* and *c* changes much slower with the volume than that of the lattice constant *b*, which indicates the weak inter-layer binding energy along the *b* direction in ZrTe_5_. The parabolic-like relationship between total energy of unit cell and volume is expected and given in Fig. 2(b). The blue vertical dotted line represent the experimental volume at low temperature (10 K), which is nearly 1.9% smaller than our calculated value.

**Figure 3:**
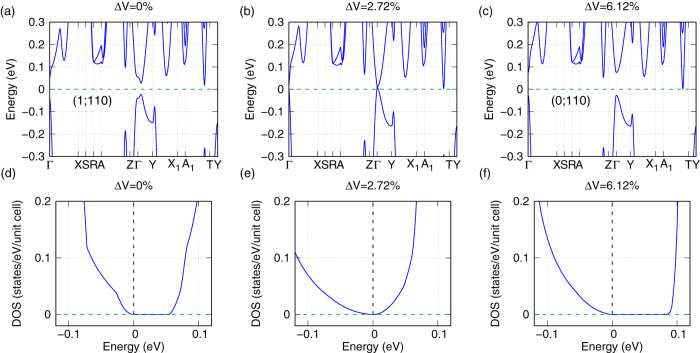
Band structures (**a–c**) and their corresponding DOSs (**d–f**) with SOC of ZrTe_5_ under different volumes. The high-symmetry points are given in Fig. 1(b). Fermi energy is set as 0.

Then we have calculated the band structures and the DOSs with spin-orbit coupling (SOC) under variant volumes, and three of them are shown in Fig. 3. The calculated band structure (Fig. 3(a)) at the ground state volume (Δ*V* = 0) is similar as previous theoretical computation^9^, although we use a different high symmetry k-path. A clear direct band gap about 94.6 meV at Γ point is found in the band structure. Of course it is also found that the valence band maxima is between the Γ and Y point and conduction band minima is between the A_1_ and T point. Therefore the indirect band gap is much smaller than the direct one at Γ point, which is about 41.7 meV in Fig. 3(a). The present of a clear band gap is confirmed in its corresponding DOS (Fig. 3(d)). Our calculated band gap is comparable with the values observed in the previous experiments, which are 80 or 100 meV^16^,^17^. We also calculated the 3D iso-energy surface (not shown here) of ZrTe_5_ in the whole Brillouin zone by the wannier functions and confirmed again that there is a global band gap in ZrTe_5_ when Δ*V* = 0%.

When ZrTe_5_ expands from its ground state, the band gap decreases gradually until the valence and conduction bands touch each other at a critical volume expansion ratio about 2.72%. Then, a Dirac point is formed at Γ point, which can be clearly seen in Fig. 3(b). This behavior is also confirmed in its corresponding V-shaped DOS near the Fermi energy, as shown in Fig. 3(e), which is the feature of Dirac point in band structure. It is noted that this Dirac point is 4-fold degenerate since ZrTe_5_ has both the space inversion and time reversal symmetry. As the crystal continues to expand, the band gap of ZrTe_5_ opens again, and ultimately reaches a value of about 102.6 (direct) or 27.7 meV (indirect) under a volume expansion 6.12%. (see Fig. 3 (c) and (f)). This band gap is also confirmed by the 3D iso-energy surface of ZrTe_5_ in the Brillouin zone. Therefore from Fig. 3, we can clearly see a transition from a semiconductor to a semi-metal and then to a semiconductor again in ZrTe_5_ when it expands. In order to check whether such a transition is topological or not, we have calculated the *Z*_2_ indices under each volume^26^. It is found that the *Z*_2_ indices are all (1;110) when the volume expansion is less than 2.72%, while it is (0;110) when the volume expansion is larger than 2.72%. This definitively confirms that ZrTe_5_ undergoes a topological phase transition from a strong TI, to an intermediate Dirac semi-metal state, and finally turns to a weak TI when its unit cell expands from 0 to 6.12% in our calculation. We noted that our calculated weak indices (110) are different from Weng’s calculation^9^ but same as Manzoni’s^19^ since the weak indices of *Z*_2_ depend on the choice of the unit cell^26^.

**Figure 4:**
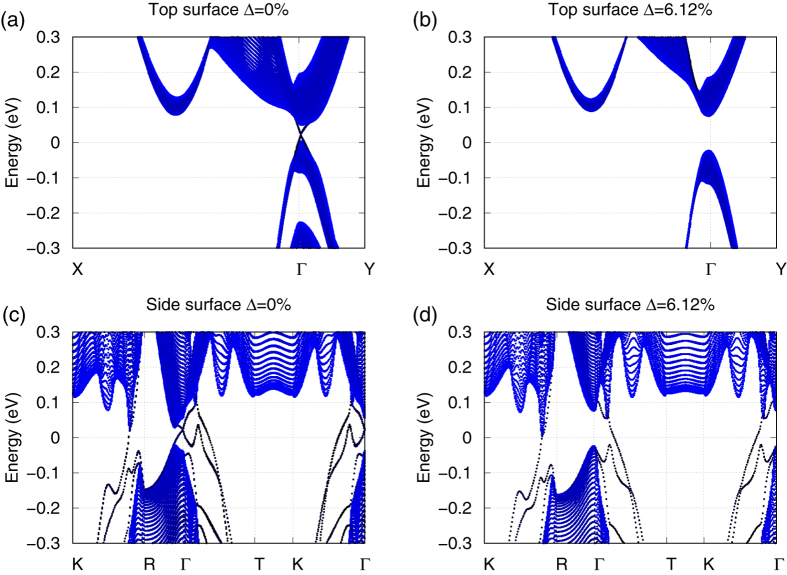
Calculated surface states of the top surface (*a*-*c* plane, i.e. cleavage surface) for the strong (**a**) and weak (**b**) TI phase. Calculated surface states of the side surface (*a*-*b* plane) for the strong (**c**) and weak (**d**) TI phase. Fermi energy is set as 0.

The surface states of ZrTe_5_ in the strong and weak TI phase have also been calculated based on the wannier functions, shown in Fig. 4. The surface band structures are very similar as the ones presented in Weng’s work^9^, since we use the similar high symmetry k-path in the surface Brillouin zone. From Fig. 4, we can see that there is a Dirac point at Γ point in top surface’s band structure for the ZrTe_5_ of Δ*V* = 0% while it does not for the case of Δ*V* = 6.12%. This key difference confirms again that ZrTe_5_ is a strong TI when Δ*V* = 0% and it becomes a weak TI when Δ*V* = 6.12%.

**Figure 5:**
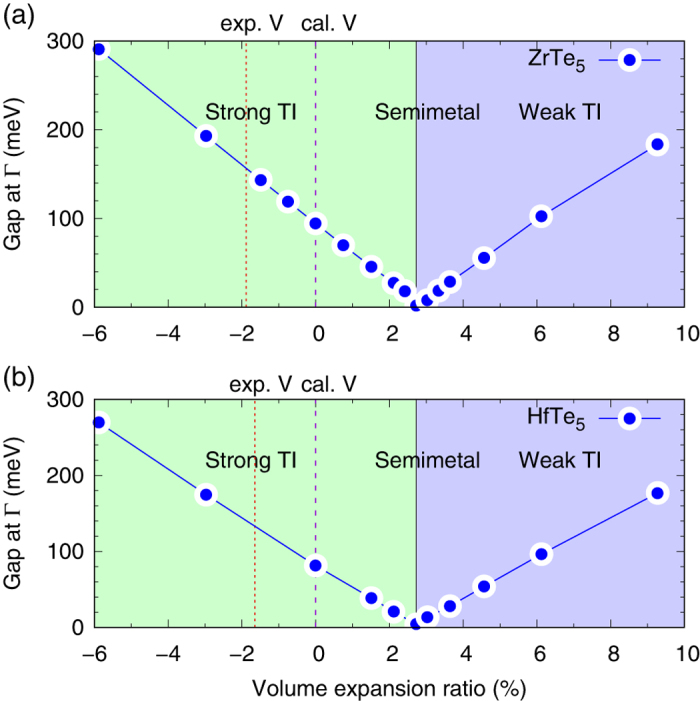
(**a**) Calculated absolute value of direct band gap at Γ point of ZrTe_5_ under different volumes. The light green and blue region represent the phases of strong and weak Tis respectively. The boundary between the strong and weak TI is the semi-metal state. The red and blue dotted vertical lines represent the experimental volume at 10 K and calculated one at ground state respectively. (**b**) Same as (**a**) but for HfTe_5_.

The detailed phase diagrams of such a topological phase transition of ZrTe_5_ and HfTe_5_ are given in Fig. 5, in which all the calculated absolute value of direct band gaps at Γ point under different volumes are plotted. In Fig. 5(a) we can find that the band gap of ZrTe_5_ decreases linearly as the volume increases from a negative volume expansion ratio about −6%, with a rate around −33 meV per 1% change of volume, where the negative value means a decrease of the band gap when the crystal expands. The band gap disappears at Δ*V* = 2.72%. Then it raises linearly with volume in a similar rate of 28 meV per 1% change of volume. Therefore ZrTe_5_ undergoes a topological transition from a strong TI to a weak TI due to volume expansion. Such a transition must need a zero-gap intermediate state, which is the Dirac semi-metal state found at about Δ*V* = 2.72% in our calculation. Similar phase diagram is also found recently by Manzoni *et al*.^19^, in which they present the band gap at Γ point as a function of the inter-layer distance, but not the volume of the unit cell. It is known that the mono-layer ZrTe_5_ is a quantum spin Hall insulator^9^. When we stack many mono-layers of ZrTe_5_ into a 3D bulk ZrTe_5_ crystal, it would be a 3D strong or weak TIs which depends on the strength of coupling between the adjacent layers. From Manzoni’s^19^ and our calculation, it is obvious that the inter-layer distance is the key factor that causes the transition between the strong and weak TI phases in ZrTe_5_. In Fig. 5(b), we also show that HfTe_5_ has the very similar topological phase transition, with almost the same transition critical volume expansion ratio at about 2.72%. The band gap of HfTe_5_ also changes linearly as the volume increases with a rate about −31 and 26 meV per 1% change of volume in the strong and weak TI region respectively.

## Discussion

The changing rate of our calculated band gap is quite significant especially in a small band gap semiconductor material. Therefore, we can conclude that the electronic properties of ZrTe_5_ (HfTe_5_) are indeed very sensitive to the change of the volume and they are indeed located very close to the boundary between the strong and weak TI. Although our optimized and the experimentally measured volume of ZrTe_5_ (HfTe_5_) both indicate that they should be within the strong TI region, we think it still has the possibility that ZrTe_5_ (HfTe_5_) can locate in a weak TI region due to different growth methods and characterization techniques in experiments. According to Fig. 5, it is even possible that ZrTe_5_ (HfTe_5_) can be very close to the intermediate Dirac semi-metal state if it happens to have a proper volume expansion ratio, which, however, is quite challenging in experiment. Another more possible reason which can explain the semi-metal behavior found in experiments is due to the defect and doping, which make the ZrTe_5_ (HfTe_5_) being a degenerate semiconductor. In a degenerate semiconductor, the Fermi energy is located within the conduction or valence band due to the doping effect, and the crystal will behave like a metal. But in this case, the energy gap still exist just below or above the Fermi energy and the Dirac point is not needed in the energy gap. This possibility is verified in a recent experimental work by Shahi *et al*. They found that the resistance anomaly of ZrTe_5_, which was observed in many existing experiments, is due to the Te deficiency, while the nearly stoichiometric ZrTe_5_ single crystal shows the normal semiconducting transport behavior^27^. In order to avoid the possible artificial effect induced by the cleavage in both STM and ARPES experiments, we suggest that nondestructive optical measurements for the existence of a direct band gap at Γ point, and its change under different temperatures, in the high quality and stoichiometric single crystals are probably useful to elucidate the topological nature in ZrTe_5_ and HfTe_5_.

Finally we show the importance of our calculated change rate of band gap. First we can roughly estimate the bulk thermal expansion coefficient from experimental lattice constants of ZrTe_5_ (Table 1) to be about 3.4 × 10^−5^ K^−1^, which means that the volume will change about 1% when the temperature changes from 0 to 300 K, equivalently the band gap of ZrTe_5_ at Γ point will change about −33 meV for strong TI phase or 28 meV for weak TI phase, according to our calculation. In a recent high-resolution ARPES work^28^, Zhang *et al*. found a clear and dramatic temperature dependent band gap in ZrTe_5_, from which we then can estimate that change rate of observed band gap is about 26 or 37 meV from 0 to 300 K depending on the methods used in their experiment. These two values are both well consistent with our calculated result. Moreover, the positive change rate found in the experiment^28^ implies that the ZrTe_5_ crystal used in their experiment is probably a weak TI according to our calculated phase diagram in Fig. 4.

In summary, we have studied the band structures of ZrTe_5_ and HfTe_5_ at variant volumes by first principles calculations. A clearly volume dependent strong and weak topological phase transition is found, accompanied by an intermediate Dirac semi-metal state at the boundary between the transition. The direct band gap of ZrTe_5_ at Γ point changes linearly with the volume, which is −33 meV and 28 meV in a strong and weak TI phase respectively, if the volume of ZrTe_5_ increases 1%, or equivalently if the temperature increases from 0 to 300 K. The results for HfTe_5_ is very similar to those of ZrTe_5_. Our calculated results indicate that the electronic properties and topological nature of ZrTe_5_ and HfTe_5_ are indeed very sensitive to the lattice constants of crystals, which is probably the reason for the divergent experimental results at present. We suggest that high quality and stoichiometric single crystal with accurate structure refinement at different temperatures would be helpful to resolve the divergent experimental results in ZrTe_5_ and HfTe_5_.

## Methods

The geometric and electronic properties of ZrTe_5_ and HfTe_5_ are calculated by the density functional theory in the generalized gradient approximation implemented in the Vienna Ab-initio Simulation Package (VASP) code^29^,^30^. The projected augmented wave method^31^,^32^ and the van der Waals (vdw) corrected optB86b-vdw functional^33^,^34^ are used. The plane-wave cutoff energy is 300 eV and the k-point mesh is 8 × 8 × 4 in the calculations. And a denser k-point mesh of 24 × 24 × 12 is used in the density of state (DOS) calculation. Spin-orbit coupling (SOC) is included in the calculation except for the structural optimization.

The theoretical ground states of ZrTe_5_ and HfTe_5_ are obtained by fully optimization of the atom positions and lattice constants, until the maximal residual force is less than 0.01 V/Å. Then we vary and fix the volumes of unit cell and still optimize the atom positions and lattice constants to study the possible topological transition in ZrTe_5_ and HfTe_5_.

The maximally-localized Wannier functions of ZrTe_5_ are fitted based on the Zr’s *d* and Te’s *p* orbitals by the Wannier90 code^35^ and then the surface states are calculated by the WannierTools^36^.

## Additional Information

**How to cite this article**: Fan, Z. *et al*. Transition between strong and weak topological insulator in ZrTe_5_ and HfTe_5_. *Sci. Rep.* **7**, 45667; doi: 10.1038/srep45667 (2017).

**Publisher's note:** Springer Nature remains neutral with regard to jurisdictional claims in published maps and institutional affiliations.

## Figures and Tables

**Table 1:** Calculated and experimental lattice constants and volumes of ZrTe_5_ and HfTe_5_ in conventional unit cell.

Material	Method	*a* (Å)	*b* (Å)	*c* (Å)	*V* (Å^3^)
ZrTe_5_	PBE	4.0490	15.772	13.845	884.17
	optB86b-vdw	4.0064	14.590	13.732	802.69
	exp. (293 K)	3.9875	14.530	13.724	795.15
	exp. (10 K)	3.9797	14.470	13.676	787.55
HfTe_5_	PBE	4.0245	15.694	13.843	874.37
	optB86b-vdw	3.9799	14.564	13.743	796.58
	exp. (293 K)	3.9713	14.499	13.729	790.51
	exp. (10 K)	3.9640	14.443	13.684	783.44
